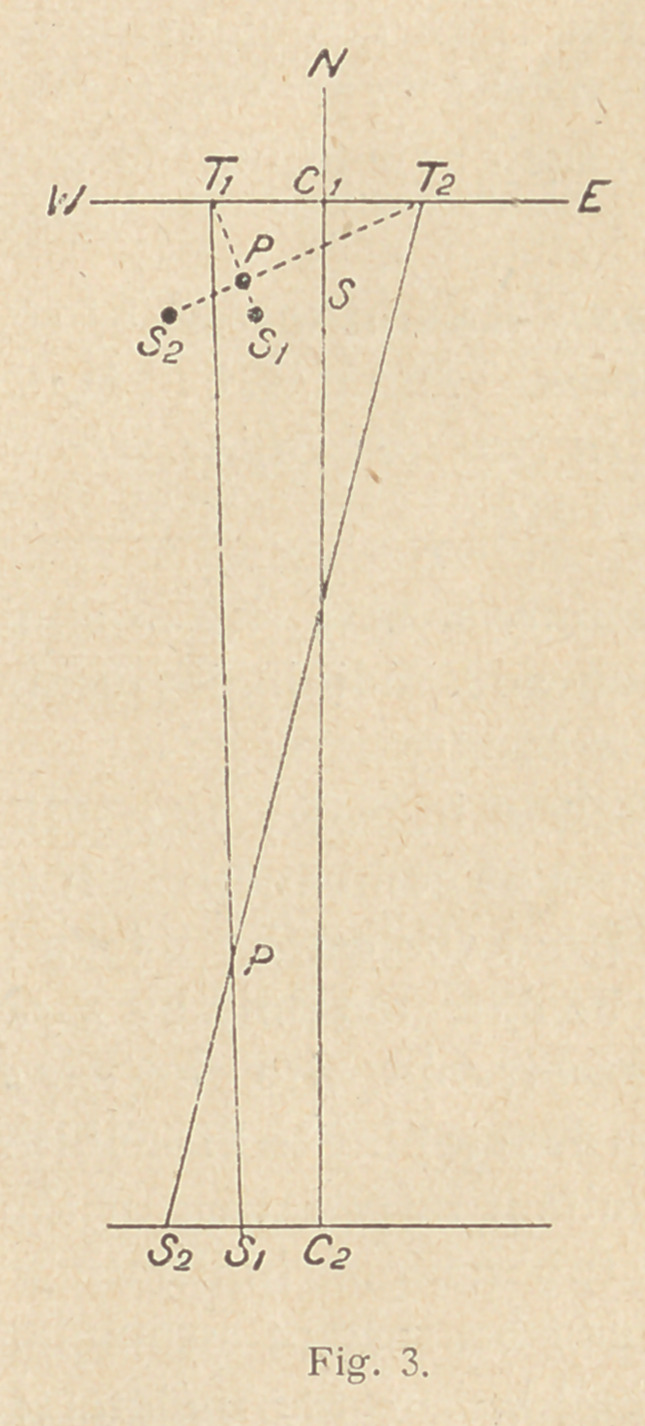# A Simple Method of X-ray Localization

**Published:** 1919

**Authors:** 


### ROENTGENOLOGY

***A Simple Method of X-ray Localization.*** By CAPTAIN D. B. MC.GRIGOR R. A. M. C. *Journal of the Royal Army Medical Corps*, September, 1918.

The following is a simple and rapid method of carrying out a complete localization without the use of any expensive apparatus.

First, make the two ordinary exposures necessary for the Mackenzie Davidson method, accurately centering your tube over the cross wires and moving the tube the usual three centimeters to each side. Both exposures can be taken on one plate, as this not only saves time and plates, but also does away with the necessity for using a plate changing box. After developing, copy the cross line markings, which are on the plate and also on the patient's skin, on a sheet of paper, as in fig. 1, along with the position of the two shadows S1 and S2.

Mark the position of the tube before and after movement T1 and T2. Join T1 and S1 and T2 and S2, the lines intersecting at P. Obviously this is just a plan view of the Mackenzie Davidson localizer, with the advantage that the point P indicating the position of

Fig. 1.

Fig. 2.

Fig. 3.

the foreign body is mathematically transferred to the plane of the negative instead of being worked out mechanically.

Second, all that is now required, besides marking point P on the patient's skin, is to find the depth of the foreign body. This can be done either by means of the well known formula $\frac{A \times S}{T + S} = D$ = depth, A being the distance of the tube to the plate, T the movement of the tube, and S the movement of the shadow; or by a simple drawing on the paper which is practically the same as the method mentioned by Dr. Hampson in the *Archives of the Roentgen Ray* for November, 1914, p. 203. This time, as the drawing (fig. 2) is obviously an elevation view of the localizer, mark the position of the two shadows $S_1$ and $S_2$, on the plate level $C_2$, $C_1$, $C_2$ being the distance that you are working at between the tube and the plate. Again join $T_1$ and $S_1$, and $T_2$ and $S_2$, which intersect at P, the elevation position of the foreign body. Now the depth D can be measured at once.

Having found the position (P in fig. 1) and the depth (D in fig. 2) of the foreign body, you have all the information required, absolutely mathematically correct, in contrast to the mechanical errors which may creep in when working with cross threads and planes.

Figs. 1 and 2 can be combined to save time as in fig. 3.

The use of N., E., S., W., for skin markings prevents any mistake occurring, such as marking the wrong quadrant on the patient's skin.

## Figures and Tables

**Fig. 1. f1:**
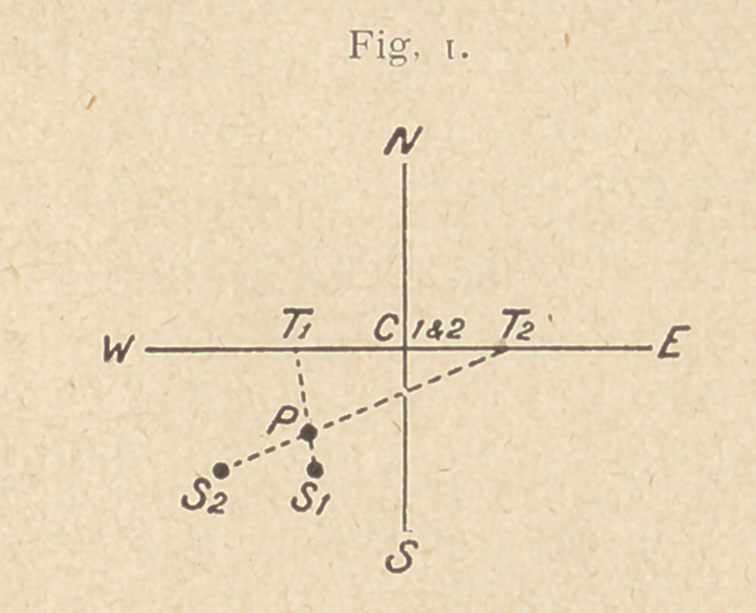


**Fig. 2. f2:**
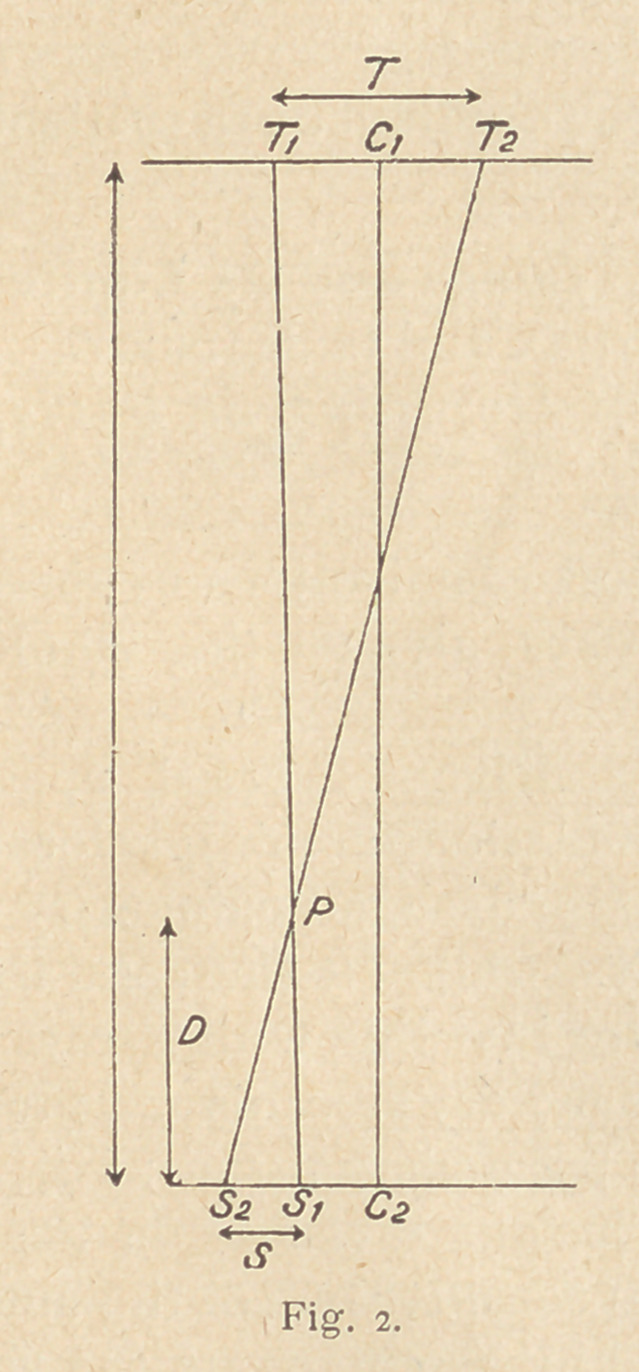


**Fig. 3. f3:**